# Adherence to drug therapy for hypertensive disorders of pregnancy: a cross-sectional survey

**DOI:** 10.1186/s13690-020-00423-0

**Published:** 2020-05-08

**Authors:** Haihong Chen, Yuqing Tang, Chenxi Liu, Junjie Liu, Kang Wang, Xinping Zhang

**Affiliations:** 1 School of Medicine and Health Management, Tongji Medical College, Huazhong University of Science and Technology, No.13. Hangkong Road, Wuhan, 430030 Hubei Province China; 2 School of Statistics and Mathematics, Central University of Finance and Economics, Beijing, China; 3 Department of Forensic Medicine, Tongji Medical College, Huazhong University of Science and Technology, Wuhan, Hubei Province China

**Keywords:** Hypertensive disorders, Pregnancy, Adherence, Drug therapy, Evidence

## Abstract

**Background:**

Hypertensive disorders of pregnancy (HDPs) are a major contributor to maternal mortality worldwide, and drug therapy for HDPs is complicated and special. Clinical guidelines help physicians optimize the care for HDPs, but little is known about whether physicians adhere to drug therapy guidelins well, especially in China. This study aims to evaluate adherence to the drug therapy guidelines of the Chinese Obstetricians and Gynecologists Association (COGA) for HDPs and to explore the corresponding associations with recommendation evidence.

**Methods:**

A cross-sectional design was executed for 306 women with HDPs hospitalized in a maternity ward of a tertiary hospital from August 2014 to July 2015 in Hubei, China. Adherence to the COGA guidelines was evaluated according to six items: the time of use and route of administration and dosage of antihypertensive drugs, MgSO_4_, and corticosteroids. Binary logistic regression was adopted to explore the associations between adherence to clinical decisions and recommendation evidence.

**Results:**

The average adherence rate for drug therapy for HDPs was 48.22%. The adherence rate for the time of antihypertensive drug and corticosteroid use scored 95.65 and 86.75%, whereas the other four items of the time of MgSO_4_ use and the routes of administration and dosages of antihypertensive drugs, MgSO_4_, and corticosteroids scored < 50.00%. High- and low-evidence-based recommendations were followed in 40.00 and 54.70% of the decisions, respectively. Logistic regression revealed that recommendation evidence (OR = 0.588, *P* = 0.003) was associated with adherence.

**Conclusions:**

Further improvement is still needed to achieve good adherence, especially regarding the time of MgSO_4_ use and drug dosage. High-evidence-based management of drug therapy for HDPs should be strengthened.

## Background

Hypertensive disorders of pregnancy (HDPs) affect approximately 5 to 10% of all pregnancies [1]. HDPs include gestational hypertension, chronic hypertension, pre-eclampsia, eclampsia, and chronic hypertension with superimposed pre-eclampsia [1–3]. HDPs are a major contributor to maternal mortality worldwide [4, 5] and are responsible for 16.1 and 9.1 to 25.7% of maternal deaths in developed and developing countries, respectively [4].

Evidence-based clinical practice guidelines (CPGs) should promote best practices in HDP management to reduce mortality in women with or at risk of developing HDPs, and to reduce infant morbidity and mortality [6]. To help physicians optimize care for HDPs, many national and international evidenced-based CPGs on HDP management, such as the guidelines of the Chinese Obstetricians and Gynecologists Association (COGA), Society of Obstetricians and Gynaecologists of Canada (SOGC), and World Health Organization (WHO), have been published [3, 7].

Drug therapy for HDPs is complicated and special because it considers the mother and the foetus. Drug therapy recommendations in guidelines involve a careful balance between risk and benefit, with an overall goal of improving maternal and foetal outcomes [8]. Adherence to these recommendations increases the use of necessary treatment and reduces maternal and perinatal risk [9, 10].

Previous studies evaluating drug therapy guideline adherence for HDPs have mainly focused on the time of drug use [11–13], and guideline adherence regarding the route of administration and drug dosage is unknown, especially in China. The associations between drug therapy guideline adherence for HDPs and recommendation evidence are also unclear.

This study aims to evaluate adherence to the COGA drug therapy guidelines for HDPs in terms of time of drug use, route of administration, and drug dosage and to explore the corresponding associations with recommendation evidence in a tertiary hospital in Hubei, China. The findings will improve drug therapy management and guideline implementation for HDPs, especially the implementation of recommendations with high evidence.

## Methods

### Design and setting

A cross-sectional design was used for the study. The study was conducted in the maternity ward of a tertiary hospital in Hubei, China. This tertiary hospital is a university teaching hospital with a 6000 bed facility, attending approximately 17,000 outpatients per day.

### Participants and sampling

The source populations were all women (*n* = 415) with HDPs hospitalized in a maternity ward at this tertiary hospital from August 2014 to July 2015. We excluded 109 women with the following conditions: (1) no specific diagnosis of an HDP or the corresponding classification (gestational hypertension, chronic hypertension, non-severe or severe pre-eclampsia, eclampsia, or chronic hypertension with superimposed non-severe or severe pre-eclampsia) based on the COGA guidelines [3]; (2) < 28 weeks of gestation; (3) no birth at this tertiary hospital; (4) stillbirth or postpartum bleeding at admission; or (5) incomplete medical records. Finally, 306 women were included in the analysis. Among them, 47 were diagnosed with gestational hypertension, 17 with chronic hypertension, 40 with non-severe pre-eclampsia, 184 with severe pre-eclampsia, and 18 with chronic hypertension with superimposed severe pre-eclampsia.

## Measurements

### Items of adherence measurement

Six items of drug therapy and nine detailed items of drug dosage were created to evaluate the drug therapy guideline adherence (Table 1), consistent with the recommendations of the COGA guidelines in 2012 [3], on which the clinical decisions of the physicians were based in our study. The translation of these recommendations of the COGA guidelines is presented in Additional file 1. We evaluated the drug therapy guideline adherence for antihypertensive drugs, MgSO_4_, and corticosteroids—the main drugs used for the management of HDPs. Each item was examined for conditionality; that is, items were considered applicable for women with underlying conditions that would have qualified for given the treatments [14]. For each item, the physicians’ treatment behaviour for every woman to whom the items were applicable was considered a clinical decision.

**Table 1:** Definitions of the six items for drug therapy of HDPs and their evidence

Items	Definitions of applicable women^a^	Definitions of adherence	Evidence
**Antihypertensive drugs**			
Q1: Time of antihypertensive drug use	Women with BP ≥ 160/110 mmHg	Use antihypertensive drugs	low
Q2: Dosage of antihypertensive drugs^b^	Women with BP ≥ 160/110 mmHg treated with oral nicardipine^c^	Adherence to per dose and dosing frequency	low
Q2–1 Per dose		Initial dose 20–40 mg	
Q2–2 Dosing frequency		Three times a day	
**MgSO**_**4**_			
Q3: Time of MgSO_4_ use	Women with severe pre-eclampsia	Use MgSO_4_	high
Q4: Route of administration and dosage of MgSO_4_	Women with severe pre-eclampsia treated with MgSO_4_	Adherence to route, loading dose, and maintenance dose	low
Q4–1 Route		Intravenous or intramuscular	
Q4–2 Loading dose		2.5–5 g	
Q4–3 Maintenance dose		Not more than 25 g in 24 h	
**Corticosteroids**			
Q5: Time of corticosteroid use	Women with severe pre-eclampsia, before 34 weeks of gestation when delivery is probable within 7 days	Use corticosteroids	high
Q6: Route of administration and dosage of corticosteroids ^b^	Women with pre-eclampsia, before 34 weeks of gestation when delivery is probable within 7 days, treated with dexamethasone	Adherence to route, per dose, dosing frequency, and continuous treatment times	low
Q6–1 Route		Intramuscular	
Q6–2 Per dose		5 mg	
Q6–3 Dosing frequency		Two times a day	
Q6–4 Continuous treatment times		Four times	

Notes: *HDPs* hypertensive disorders of pregnancy. Qi (i = 1 to 6) is the code for items, and Qi-j (i = 2, 4 or 6; j = 1,2,3 or 4) is the code for detailed items of Qi

^a^ The applicable and adhering situation of women with chronic hypertension with superimposed pre-eclampsia referred to women with chronic hypertension or pre-eclampsia [3]. ^b^ In the route of administration and dosage of antihypertensive drugs and corticosteroids, the most frequently used drugs, such as nicardipine and dexamethasone, are presented. ^c^ Data on the dosage of intravenous nicardipine were unavailable. Hence, only the dosage of oral nicardipine was evaluated

### Indicators of adherence measurement

The primary indicator used for evaluating the adherence to drug therapy was the adherence rate, and secondary indicators were underuse and overuse rates. The adherence rate for each item was calculated as the sum of the number of clinical decisions for the item where the physician adhered to the guidelines divided by the number of clinical decisions for the item. The overall adherence rate for all the items was calculated as the sum of the number of clinical decisions for all items where physicians adhered to the guidelines divided by the number of clinical decisions for all the items. The underuse/overuse rate for each item was calculated as the sum of the number of clinical decisions that physicians treated with the drugs at a dosage that was less/more than that recommended for the item divided by the number of clinical decisions for this item. Six items of drug therapy and nine detailed items of drug dosage could be calculated by the adherence rate, but underuse and overuse rates could only be calculated for nine detailed items of drug dosage.

### Recommendation evidence measurements

In the COGA guidelines, some recommendations were ascribed with evidence levels *I, II-1, II-2, II-3,* or *III*. These evidence levels were defined in accordance with the guidelines of the SOGC [15]. For example, level *I* indicates that evidence was obtained from at least one properly randomized controlled trial. In this study, we considered level *I* as high evidence and *II-1, II-2,* and *II-3* as moderate evidence. Level *III* and without evidence or references behind recommendations were considered low evidence. The evidence levels for the six items of drug therapy are listed in Table 1. We found that none of six items was moderate evidence according to Chinese guidelines, so the evidence levels of these items were either high or low.

#### Data collection

Data on women’s general characteristics (type of HDPs, weeks of gestation at admission, type of delivery, number of foetuses, time from admission to delivery, health insurance, age, parity, and number of abortions), blood pressure with multiple monitoring, and drug therapy from admission to delivery were collected by electronic medical records. The records are represented by International Classification of Diseases, Tenth Revision (ICD 10) codes.

#### Statistical analysis

Descriptive statistics included frequencies and proportions for categorical data and median with lower quartile (P_25_) and upper quartile (P_75_) for continuous data. To explore the association between guideline adherence and recommendation evidence, we performed binary logistic regression by using adherence to clinical decisions (*n* = 647) as the dependent variable, and recommendation evidence and general characteristics as the independent variables. Standard error was clustered at the patient level. In the logistic regression, the analytical unit was each item (Q1, Q2, Q3, Q4, Q5 or Q6) that did not include nine detailed items for every woman, and the overall number of clinical decisions for 306 women was 647. Each item could be evaluated with whether adhered to, but underuse or overuse could only be applicable to drug dosage which was the details of Q2, Q4 or Q6. Therefore, we did not use underuse or overuse as the dependent variable in the logistic regression. We used the Pearson chi-square goodness-of-fit test to assess the goodness of fit of our model. Odds ratios (ORs) and 95% confidence intervals (CIs) were calculated. *P* < 0.05 was considered statistically significant. Statistical analysis was conducted using STATA 12.0 (STATA Corp, College Station, TX, USA).

## Results

### General characteristics of participants

The characteristics of the 306 women are summarized in Table 2. The majority of the women were diagnosed with severe pre-eclampsia (60.13%), were admitted at ≥37 weeks of gestation (38.56%), underwent caesarean delivery (95.75%), carried a single foetus (83.99%), spent ≥24 h from admission to delivery (68.95%), and possessed health insurance (89.87%). The median age, parity, and number of abortions of these women were 30 years, zero, and one, respectively.

**Table 2:** General characteristics of women with HDPs

Variable name	Frequency (percentage)/median(P_25_ ~ P_75_)^a^*N* = 306
Type of hypertensive disorders of pregnancy, n(%)	
Gestational hypertension	47 (15.36)
Chronic hypertension	17 (5.56)
Non-severe pre-eclampsia	40 (13.07)
Severe pre-eclampsia	184 (60.13)
Chronic hypertension superimposed severe pre-eclampsia	18 (5.88)
Weeks of gestation at admission, n(%)	
28~	91 (29.74)
34~	97 (31.70)
37~	118 (38.56)
Type of delivery, n(%)	
Eutocia	10 (3.27)
Caesarean	293 (95.75)
Induced labour	3 (0.98)
Number of foetuses, n(%)	
Single	257 (83.99)
Twins	49 (16.01)
The time from admission to delivery, n(%)	
< 24 h	95 (31.05)
≥ 24 h	211 (68.95)
Health insurance, n(%)	
No	31 (10.13)
Yes	275 (89.87)
Age, median(P_25_ ~ P_75_)	30 (27 ~ 35)
Parity, median(P_25_ ~ P_75_)	0 (0 ~ 1)
Number of abortions, median(P_25_ ~ P_75_)	1 (0 ~ 2)

Notes: *HDPs* hypertensive disorders of pregnancy

^a^ P_25_ representes the lower quartile, and P_75_ representes the upper quartile

### Adherence, underuse and overuse rates

The average adherence rate for drug therapy for HDPs was 48.22% (312/647), ranging from 0 to 95.65% for six items. High- and low-evidence-based recommendations were followed in 40.00% (114/285) and 54.70% (199/362) of decisions, respectively.

**Fig. 1:**
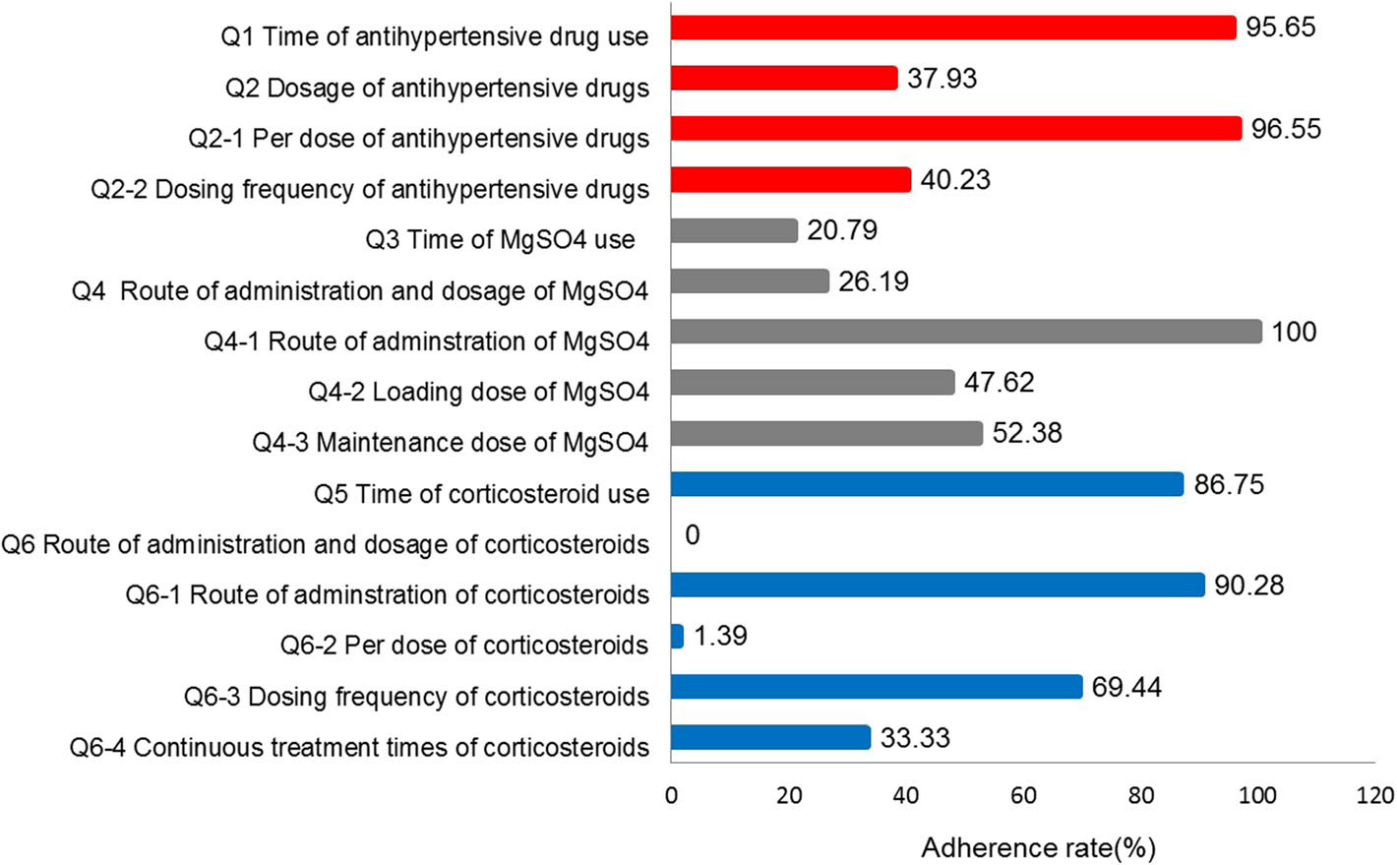
Adherence rates for antihypertensive drugs, MgSO4, and corticosteroids. Qi (i = 1 to 6) is the code for items, and Qi-j (i = 2, 4 or 6; j = 1,2,3 or 4) is the code for detailed items of Qi. The red, grey and blue rectangles represent the adherence rates for antihypertensive drugs, MgSO4 and corticosteroids, respectively

The adherence rates for antihypertensive drugs, MgSO_4_, and corticosteroids are presented in Fig. 1. The adherence rates for the time of antihypertensive drug and corticosteroid use were 95.65 and 86.75%, respectively. However, the adherence rate for the time of MgSO_4_ use scored 20.79%, which was relatively poor. Although the adherence rates for the routes of administration of MgSO_4_ and corticosteroids were 100 and 90.28%, respectively, adherence to the dosages of antihypertensive drugs, MgSO_4_, and corticosteroids were relatively poor.

**Fig. 2:**
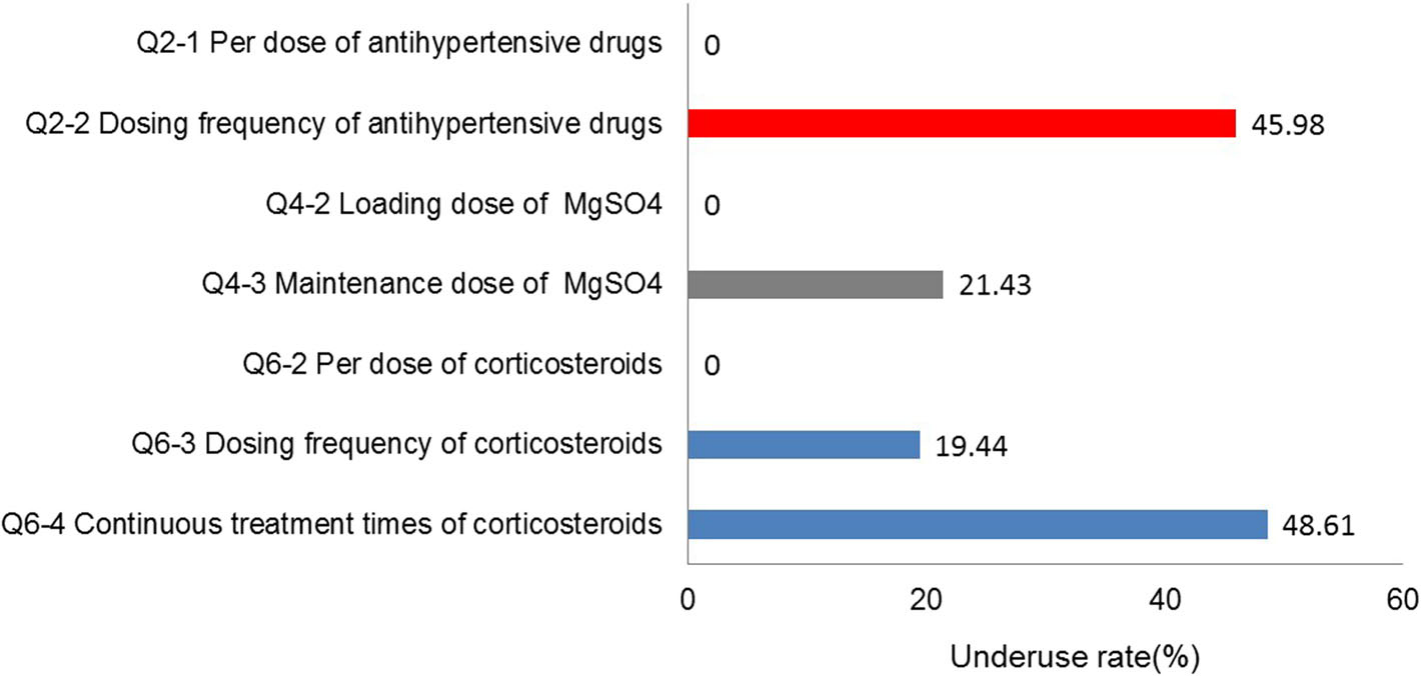
Underuse rates for antihypertensive drugs, MgSO4, and corticosteroids. Qi-j (i = 2, 4 or 6; j = 1,2,3 or 4) is the code for detailed items of dosage of drugs. The red, grey and blue rectangles represent the underuse rates for antihypertensive drugs, MgSO4 and corticosteroids, respectively

**Fig. 3:**
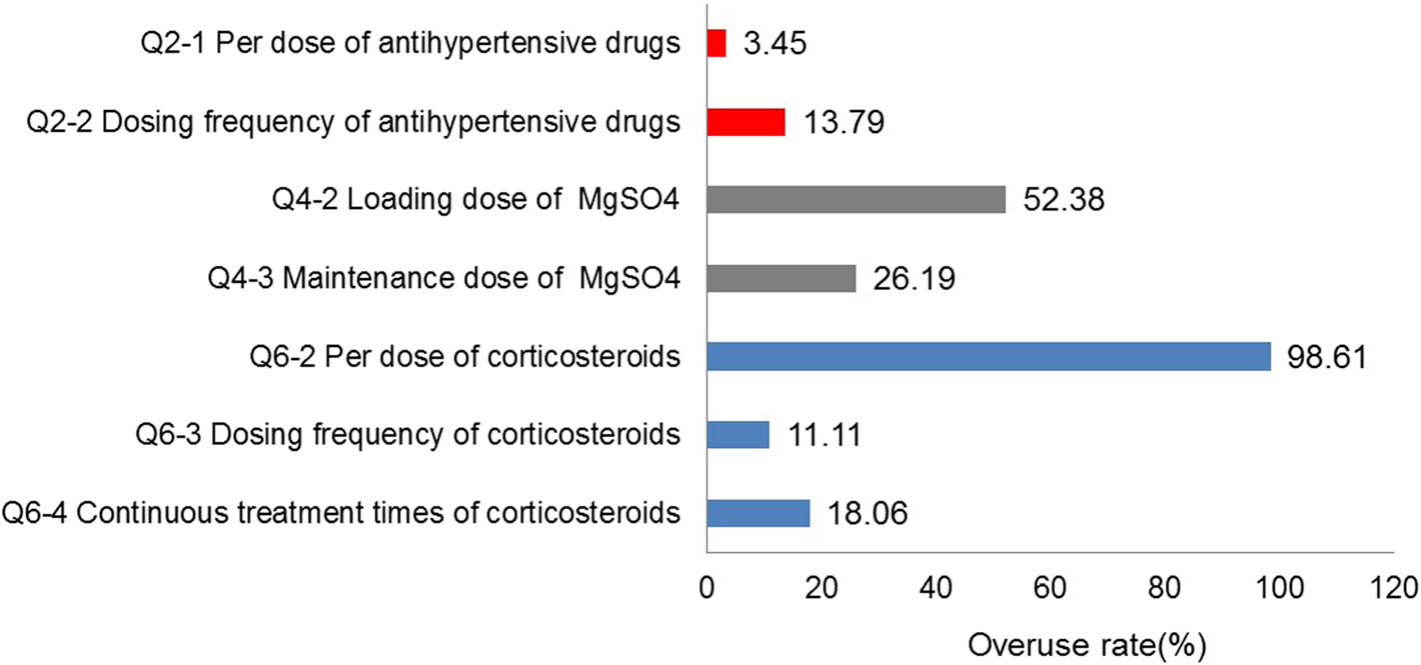
Overuse rates for antihypertensive drugs, MgSO4 and corticosteroids. Qi-j (i = 2, 4 or 6; j = 1, 2, 3 or 4) is the code for detailed items of the dosage of drugs. The red, grey and blue rectangles represent the overuse rates for antihypertensive drugs, MgSO4 and corticosteroids, respectively

The underuse and overuse rates for these drugs are presented in Figs. 2 and 3, respectively. No women were treated with an inadequate loading dose of MgSO_4_ or inadequate per dose of antihypertensive drugs or corticosteroids, and 45.98% were treated with an inadequate dosing frequency of antihypertensive drugs. However, the overuse rate for the loading dose of MgSO_4_ was 52.38%, and those of the per dose, dosing frequency and continuous treatment times of corticosteroids were 98.61, 11.11 and 18.06%, respectively. Details on the guideline adherence regarding antihypertensive drugs, MgSO_4_, and corticosteroids are presented in Additional files 2, 3 and 4: Tables S1, S2 and S3.

### Association between adherence and recommendation evidence

The logistic regression analysis results are shown in Table 3. The recommendation evidence (OR = 0.588, *P* = 0.003) was significantly associated with guideline adherence.

**Table 3:** Binary regression analysis

	z	standard error	*P*	OR	95% CI	
					Lower	Upper
Type of hypertensive disorders of pregnancy						
Severe pre-eclampsia	−2.57	0.084	**0.010**	0.751	0.604	0.934
Non-severe pre-eclampsia^a^	_	_	_	_	_	_
Chronic hypertension	1.38	1.503	0.166	2.389	0.696	8.199
Gestational hypertension	1.10	1.066	0.271	1.872	0.613	5.717
Weeks of gestation at admission						
34~	0.70	0.180	0.487	1.119	0.816	1.534
37~	2.20	0.188	**0.028**	1.357	1.033	1.781
Type of delivery						
Caesarean	2.30	0.365	**0.021**	1.659	1.078	2.555
Induced labour	0.48	0.414	0.635	1.181	0.595	2.346
Number of foetuses	0.84	0.159	0.403	1.125	0.853	1.484
Time from admission to delivery	1.02	0.133	0.309	1.127	0.895	1.420
Health insurance	−0.81	0.142	0.418	0.877	0.639	1.204
Age	−0.11	0.010	0.916	0.999	0.979	1.019
Parity	0.39	0.087	0.699	1.033	0.876	1.218
Number of abortions	0.72	0.040	0.473	1.028	0.953	1.108
Evidence	−2.94	0.106	**0.003**	0.588	0.412	0.838
Constant	−1.28	0.221	0.201	0.646	0.331	1.262
Goodness-of-fit test^b^			0.974			

Notes: ^a^ In the logistic regression, the number of non-severe pre-eclampsia cases was only 2, so the z value, standard error, *P* value, OR and 95% CI were not estimated

^b^ Pearson Chi-Squared goodness-of-fit test

## Discussion

In this study on drug therapy guideline adherence for HDPs, we found that the average adherence rate for drug therapy for HDPs is not optimistic (< 50%), especially regarding the time of MgSO_4_ use and drug dosage. High adherence is negatively associated with high recommendation evidence.

The adherence rate for time of MgSO4 use was 20.79%. Similar findings have been previously reported [12, 13]. However, Shields et al. showed that high levels of compliance can be achieved in a relatively short period of time [16]. Thus, active measures, such as providing feedback on the quality of care, drilling and displaying guidelines, and using web-based applications to improve adherence on the time of MgSO_4_ use must be employed [12, 17].

This study revealed that guideline adherence regarding drug dosage was relatively low. We found that 45.98% of the women were treated with oral nicardipine fewer than three times a day, and 52.38% of the women were treated with an overstandard loading dose of MgSO_4_. These findings highlighted that the dosing frequency of oral nicardipine and loading dose of MgSO_4_ were overlooked. The overuse of per dose, dosing frequency and continuous treatment times of dexamethasone were serious problems. The use of repeated courses of antenatal corticosteroids was one of the issues [18], and dexamethasone has been overused in many clinical departments, such as emergency [19] and paediatrics [20]. Overusing corticosteroids antenatally does not improve outcomes and is associated with increased mortality, decreased foetal growth, and prolonged adrenal suppression [21].

Notably, the adherence to high evidence-based recommendations was low. Grol et al. found that general practitioners adhered to more evidence-based recommendations than those not based on evidence [22]. This difference was probably associated with different study populations. We mainly focused on obstetricians in a tertiary hospital rather than general practitioners. Although the evidence for the recommendations was essential [22], high evidence was not always associated with high adherence. First, clinical evidence for drug therapy for HDPs was relatively absent in general, and some recommendations were based more on opinion than on evidence [23]. For example, antihypertensive treatment was strongly recommended by the WHO for severe hypertension [23]. The adherence rate of this recommendation was high in our study despite low scientific evidence. Second, some high-evidence-based recommendations, especially the time of MgSO_4_ use, were overlooked in clinical practice. Finally, the clinical conditions were highly complicated, and factors associated with guideline adherence were complex. Abnormal laboratory or physical findings, high operative risk, intolerance for recommended treatment, and extensive comorbidities may influence guideline adherence [24], causing poor adherence of physicians to high-evidence-based recommendations. The sample size for the logistic analysis in our study was 647 (adherence and nonadherence were 312 and 335, respectively), and the overall adherence rate was 48.22%. The number of independent variables was 10. According to the rule of thumb that logistic models should be used with a minimum of 10 events per variable [25], we estimate that the minimum sample size should be 208 (10 ∗ 10/48.22 %). Therefore, we thought the sample size might be sufficient for the logistic analysis.

There were some limitations to this study. First, physician factors, such as unfamiliarity with the guidelines [26], lack of guideline awareness [27], and difficulty changing routines and habits [28], may cause low adherence. However, professional factors have not been included as an independent variable in the regression analysis for unavailable data. Second, adherence to guidelines is professional-specific, and different physicians work in different contexts, so our results might not be transferable to other settings. In addition, we excluded women with incomplete medical records which may lead to selection bias.

## Conclusions

Further improvement is still needed to achieve good adherence, especially regarding the time of MgSO_4_ use and drug dosage. High-evidence-based management for drug therapy for HDPs should be strengthened. Improvement in drug therapy with high-evidence will promote rational use of drugs and reduce maternal and perinatal risk.

## Supplementary information

**Additional file 1.**

**Additional file 2: Table S1.** Guideline adherence for antihypertensive drugs.

**Additional file 3: Table S2.** Guideline adherence for MgSO_4_.

**Additional file 4: Table S3.** Guideline adherence for corticosteroids.

## Data Availability

The datasets used and/or analysed during the current study are available from the corresponding author on reasonable request.

## Abbreviations

HDPs: Hypertensive disorders of pregnancy
CPGs: Clinical practice guidelines
COGA: Chinese Obstetricians and Gynecologists Association
SOGC: Society of Obstetricians and Gynaecologists of Canada
WHO: World Health Organization
ICD 10: International Classification of Diseases, Tenth Revision
OR: Odds ratios
CIs: Confidence intervals
