# Supplementary material for: Adherence to drug therapy for hypertensive disorders of pregnancy: a cross-sectional survey
Source: Arch Public Health. 2020 May 8;78:41. doi: 10.1186/s13690-020-00423-0 (PMC7206801; doi:10.1186/s13690-020-00423-0)
Supplement: Supplementary file 1 — Additional file 1. [file 13690_2020_423_MOESM1_ESM.doc]

**Additional file 1**

**Antihypertensive drugs**

Women with systolic pressure≥160 mmHg or diastolic pressure≥110 mmHg during pregnancy should receive treatment with antihypertensive drugs.

Usage of Nicardipine: (1)Oral administration: Initial dose 20–40 mg, three times a day. (2) Intravenous administration: Initial dose 1 mg/h, adjust the dose every 10 minutes according to the blood pressure.

**MgSO_4_**

MgSO_4_ is recommended for the treatment of women with eclampsia or prevention of eclampsia in women with severe pre-eclampsia in preference to other anticonvulsants.

Usage of MgSO4: (1) Treatment of women with eclampsia: Loading dose 2.5–5 g,intravenous administration or intramuscular injection before sleep at night, and 25-30 g in 24 h. (2) Prevention of eclampsia in women with severe pre-eclampsia: Loading dose 2.5–5 g, intravenous administration, and no more than 25 g in 24 h.

**Corticosteroids**

Women with severe preeclampsia, before 34 weeks of gestation when delivery is probable within 7 days should use corticosteroids for lung maturation.

Usage of Corticosteroids: (1) Dexamethasone: per dose 5 mg, intramuscular injection, two times a day, and continuous for two days; (2) Betamethasone: per dose 12 mg, intramuscular injection, one time a day, and continuous for two days.
