# Supplementary material for: Adherence to drug therapy for hypertensive disorders of pregnancy: a cross-sectional survey
Source: Arch Public Health. 2020 May 8;78:41. doi: 10.1186/s13690-020-00423-0 (PMC7206801; doi:10.1186/s13690-020-00423-0)
Supplement: Supplementary file 2 — Additional file 2: Table S1. Guideline adherence for antihypertensive drugs. [file 13690_2020_423_MOESM2_ESM.doc]

Table S1. Guideline adherence for antihypertensive drugs

|  | Gestational hypertension,  %(ni/Ni) | Chronic hypertension,  %(ni/Ni) | Non-severe pre-eclampsia  %(ni/Ni) | Severe pre-eclampsia,  %(ni/Ni) | Superimposed severe pre-eclampsia,  %(ni/Ni) | Hypertensive disorders of pregnancy,  %(ni/Ni) |
| --- | --- | --- | --- | --- | --- | --- |
| **Adherence rate** |  |  |  |  |  |  |
| Q1:Time of antihypertensive drug use | 100.00  (4/4) | 85.71  (6/7) | 100.00  (1/1) | 95.45  (126/132) | 100.00  (17/17) | **95.65**  **(154/161)** |
| Q2: Dosage of antihypertensive drugs | 33.33  (1/3) | 60.00  (3/5) | 100.00  (1/1) | 34.72  (25/72) | 50.00  (3/6) | **37.93**  **(33/87)** |
| Q2-1 Per dose | 100.00  (3/3) | 100.00  (5/5) | 100.00  (1/1) | 97.22  (70/72) | 83.33  (5/6) | 96.55  (84/87) |
| Q2-2 Dosing frequency | 33.33  (1/3) | 60.00  (3/5) | 100.00  (1/1) | 36.11  (26/72) | 66.67  (4/6) | 40.23  (35/87) |
| **Underuse rate** |  |  |  |  |  |  |
| Q2-1 Per dose | 0.00  (0/3) | 0.00  (0/5) | 0.00  (0/1) | 0.00  (0/72) | 0.00  (0/6) | 0.00  (0/87) |
| Q2-2 Dosing frequency | 66.67  (2/3) | 40.00  (2/5) | 0.00  (0/1) | 48.61  (35/72) | 16.67  (1/6) | 45.98  (40/87) |
| **Overuse rate** |  |  |  |  |  |  |
| Q2-1 Per dose | 0.00  (0/3) | 0.00  (0/5) | 0.00  (0/1) | 2.78  (2/72) | 16.67  (1/6) | 3.45  (3/87) |
| Q2-2 Dosing frequency | 0.00  (0/3) | 0.00  (0/5) | 0.00  (0/1) | 15.28  (11/72) | 16.67  (1/6) | 13.79  (12/87) |

Note: Qi (i=1 or 2) is the code for items, and Q2-j (j=1 or 2) is the code for detailed items of Q2.
