# Supplementary material for: Adherence to drug therapy for hypertensive disorders of pregnancy: a cross-sectional survey
Source: Arch Public Health. 2020 May 8;78:41. doi: 10.1186/s13690-020-00423-0 (PMC7206801; doi:10.1186/s13690-020-00423-0)
Supplement: Supplementary file 3 — Additional file 3: Table S2. Guideline adherence for MgSO4. [file 13690_2020_423_MOESM3_ESM.doc]

Table S2. Guideline adherence for MgSO_4_

|  | Severe pre-eclampsia,  %(ni/Ni) | Superimposed severe pre-eclampsia,  %(ni/Ni) | Hypertensive disorders of pregnancy,  %(ni/Ni) |
| --- | --- | --- | --- |
| **Adherence rate** |  |  |  |
| Q3:Time of MgSO_4_ use | 20.11(37/184) | 27.78(5/18) | **20.79(42/202)** |
| Q4: Route of administration and dosage of MgSO_4_ | 29.73(11/37) | 0.00(0/5) | **26.19(11/42)** |
| Q4-1 Route | 100.00(37/37) | 100.00(5/5) | 100.00(42/42) |
| Q4-2 Loading dose | 48.65(18/37) | 40.00(2/5) | 47.62(20/42) |
| Q4-3 Maintenance dose | 56.76(21/37) | 20.00(1/5) | 52.38 (22/42) |
| **Underuse rate** |  |  |  |
| Q4-2 Loading dose | 0.00(0/37) | 0.00(0/5) | 0.00(0/42) |
| Q4-3 Maintenance dose | 16.22(6/37) | 60.00(3/5) | 21.43(9/42) |
| **Overuse rate** |  |  |  |
| Q4-2 Loading dose | 51.35(19/37) | 60.00(3/5) | 52.38(22/42) |
| Q4-3 Maintenance dose | 27.03(10/37) | 20.00(1/5) | 26.19 (11/42) |

Note: Qi (i=3 or 4) is the code for items, and Q4-j (j=1, 2 or 3) is the code for detailed items of Q4. Underuse of the maintenance dose of MgSO_4_ in this study means without maintenance.
