# Supplementary material for: Adherence to drug therapy for hypertensive disorders of pregnancy: a cross-sectional survey
Source: Arch Public Health. 2020 May 8;78:41. doi: 10.1186/s13690-020-00423-0 (PMC7206801; doi:10.1186/s13690-020-00423-0)
Supplement: Supplementary file 4 — Additional file 4: Table S3. Guideline adherence for corticosteroids. [file 13690_2020_423_MOESM4_ESM.doc]

Table S3. Guideline adherence for corticosteroids

|  | Severe pre-eclampsia,  %(ni/Ni) | superimposed severe pre-eclampsia,  %(ni/Ni) | Hypertensive disorders of pregnancy,  %(ni/Ni) |
| --- | --- | --- | --- |
| **Adherence rate** |  |  |  |
| Q5:Time of corticosteroid use | 85.53(65/76) | 100.00(7/7) | **86.75(72/83)** |
| Q6: Route of administration and dosage of corticosteroids | 0.00(0/65) | 0.00(0/7) | **0.00(0/72)** |
| Q6-1 Route | 90.77(59/65) | 85.71(6/7) | 90.28(65/72) |
| Q6-2 Per dose | 1.54(1/65) | 0.00(0/7) | 1.39(1/72) |
| Q6-3 Dosing frequency | 70.77(46/65) | 57.14(4/7) | 69.44(50/72) |
| Q6-4 Continuous treatment times | 33.85(22/65) | 28.57(2/7) | 33.33(24/72) |
| **Underuse rate** |  |  |  |
| Q6-2 Per dose | 0(0/65) | 0.00(0/7) | 0(0/72) |
| Q6-3 Dosing frequency | 18.46(12/65) | 28.57(2/7) | 19.44(14/72) |
| Q6-4 Continuous treatment times | 46.15(30/65) | 71.43(5/7) | 48.61(35/72) |
| **Overuse rate** |  |  |  |
| Q6-2 Per dose | 98.46(64/65) | 100.00(7/7) | 98.61(71/72) |
| Q6-3 Dosing frequency | 10.77(7/65) | 14.29(1/7) | 11.11(8/72) |
| Q6-4 Continuous treatment times | 20.00(13/65) | 0.00(0/7) | 18.06(13/72) |

Note: Qi (i=5 or 6) is the code for items, and Q6-j (j=1, 2, 3 or 4) is the code for detailed items of Q6.
